# CADM1, MAL, and miR124 Promoter Methylation as Biomarkers of Transforming Cervical Intrapithelial Lesions

**DOI:** 10.3390/ijms20092262

**Published:** 2019-05-07

**Authors:** Marta del Pino, Adriana Sierra, Lorena Marimon, Cristina Martí Delgado, Adriano Rodriguez-Trujillo, Esther Barnadas, Adela Saco, Aureli Torné, Jaume Ordi

**Affiliations:** 1 Institute Clinic of Gynecology, Obstetrics, and Neonatology, Hospital Clínic, Institut d’Investigacions Biomèdiques August Pi i Sunyer (IDIBAPS), Universitat de Barcelona, 08036 Barcelona, Spain; mdelpino@clinic.cat (M.d.P.); marti@clinic.cat (C.M.D.); adrodrig@clinic.cat (A.R.-T.); atorne@clinic.cat (A.T.); 2 Department of Pathology, Hospital Clínic, University of Barcelona, 08036 Barcelona, Spain; AdriSierra300@hotmail.com (A.S.); lmarimon@clinic.cat (L.M.); esther.barnadas@idibaps.org (E.B.); masaco@clinic.cat (A.S.); 3 ISGlobal, Hospital Clínic, Universitat de Barcelona, 08036 Barcelona, Spain

**Keywords:** CADM1, MAL, miR124, methylation, HSIL

## Abstract

Background: Squamous intraepithelial lesions/cervical intraepithelial neoplasias (SIL/CIN) are high-risk human papilloma virus (hrHPV)-related lesions which are considered as high grade (HSIL/CIN2-3) or low grade (LSIL/CIN1) lesions according to their risk of progression to cervical cancer (CC). Most HSIL/CIN2-3 are considered as transforming hrHPV infections, so truly CC precursors, although some clear spontaneously. hrHPV testing has a high sensitivity for the detection of HSIL/CIN2-3 but a relatively low specificity for identifying transforming lesions. We aimed to determine whether the combination of CADM1, MAL and miR124 promoter methylation status assessed in histological samples can be used as a biomarker in the identification of transforming HSIL/CIN lesions. Design: 131 cervical biopsies, including 8 cases with no lesion and a negative hrHPV test result (control group), 19 low-grade (L)SIL/CIN1, 30 HSIL/CIN2, 60 HSIL/CIN3, and 14 CC were prospectively collected. hrHPV was detected and genotyped using the polymerase chain reaction (PCR)-based technique SPF10 HPV LIPA. A multiplex quantitative methylation-specific PCR (qMSP) was used to identify the methylation status of the CADM1, MAL, and miR124 promoter genes. Results: Significantly higher methylation levels of CADM1, MAL and miR-124 were found in HSIL/CIN2-3 and CC compared with normal and LSIL lesions. DNA methylation of at least one gene was detected in 12.5% (1/8) of normal samples, 31.5% (6/19) of LSIL/CIN1, 83.3% (25/30) of HSIL/CIN2, 81.6% (49/60) of HSIL/CIN3 and 100% (14/14) of CC (*p* < 0.001). The sensitivity and specificity for HSIL/CIN2-3 and CC of having at least one methylated gene were 84.6% and 74.0%, respectively. The sensitivity and specificity of the combination of at least one methylated gene and a positive hrHPV test were 80.7% and 85.1% for HSIL/CIN2-3 and CC, respectively. Conclusions: The methylation rate of CADM1, MAL and miR124 increases with the severity of the lesion. Further research is warranted to evaluate the usefulness of these biomarkers for the identification of transforming HSIL/CIN.

## 1. Introduction

Virtually all cervical cancers (CC) result from persistent infection by high-risk human papilloma virus (hrHPV) and are preceded by precursor lesions referred to as squamous intraepithelial lesions or cervical intraepithelial neoplasia (SIL/CIN) [1]. These precursor lesions are divided into “productive” and “transforming” lesions according to their risk of progression to CC [2]. From the clinical perspective, only the latter lesions should be considered as true premalignant abnormalities requiring exhaustive colposcopy and treatment to prevent CC. Most low-grade SIL/CIN grade 1 (LSIL/CIN1) are considered to represent productive lesions, whereas most HSIL/CIN grade 2 and 3 (HSIL/CIN2-3) are classified as transforming lesions. Nevertheless, HSIL/CIN2-3, and particularly HSIL/CIN2, is believed to be a heterogeneous disease, as suggested by the fact that some molecular features common to almost all CC are found in only a subset of HSIL/CIN2-3 [3,4,5,6], which indicates that not all these lesions have the same risk of progression to CC, and that some are productive infections. Similarly, a small proportion of LSIL may represent an early phase of a transforming lesion.

hrHPV tests have a high sensitivity for the detection of cervical premalignant lesions and CC. However, hrHPV tests have shown a low specificity and positive predictive value (PPV), as all HSIL/CIN2-3 as well as most productive LSIL/CIN1 lesions are positive for hrHPV independently of their risk of progression [7]. Thus, there is a need for biomarkers able to specifically identify the transforming HSIL/CIN2-3 lesions requiring treatment and to exclude LSIL/CIN1 and ideally the subset of HSIL/CIN2-3 thought to be productive lesions with a low risk of progression to CC, in order to reduce the adverse events and costs associated with unnecessary treatments.

Hypermethylation of CpG islands in the promoter regions of tumor suppressor genes leads to silencing of the gene and is an essential step in the development of many cancers, including CC [8]. Hypermethylation of promoter regions in certain genes has also been suggested as a molecular alteration appearing in transforming HSIL/CIN2-3 lesions at high risk of progression to CC [9,10,11,12,13,14]. Recent studies have shown that the combination of methylation-mediated silencing of CADM1 (cell adhesion molecule 1) and MAL (T-lymphocyte maturation-associated protein) promoter genes in cervical scrapes seems to be related to the duration of hrHPV infection and the severity of the SIL/CIN lesion [10,15]. In addition, methylation of CpG-rich regulatory sequences can result in a down-regulation of micro RNAs (miR) and, interestingly, down-regulation of certain miR, such as miR124, has been linked to increased promoter methylation of the CADM1 and MAL genes [16].

In the present study, we analyzed CADM1, MAL and miR124 methylation status in addition to hrHPV testing and genotyping in a series of cervical biopsies from women referred to colposcopy due to an abnormal screening test. The aim of the study was to determine if these biomarkers might be related to lesion severity and might, therefore, be useful in the characterization of transforming HSIL/CIN lesions in a referral population.

## 2. Results

One hundred forty-six histological samples were eligible for the study. Fifteen (10.27%) were excluded due to low DNA quality for methylation analysis. Of the remaining 131 samples considered as adequate for analysis, eight were negative (control group); 19 were LSIL/CIN1, 30 were HSIL/CIN2, 60 were HSIL/CIN3, and 14 were CC. The median age of the patients in each diagnostic category was 36.1 years in the control group (range 21–50 years); 36.8 years in the LSIL/ CIN1 group (range 17–60 years), 34.0 years in the HSIL/CIN2 (range 22–45 years), 36.0 years in the HSIL/CIN3 (range 23–52 years), and 53.0 years in the CC group (range 27–76 years). Women with CC were significantly older than those in the other groups (*p* < 0.001).

### 2.1. Methylation Status

**Figure 1:**
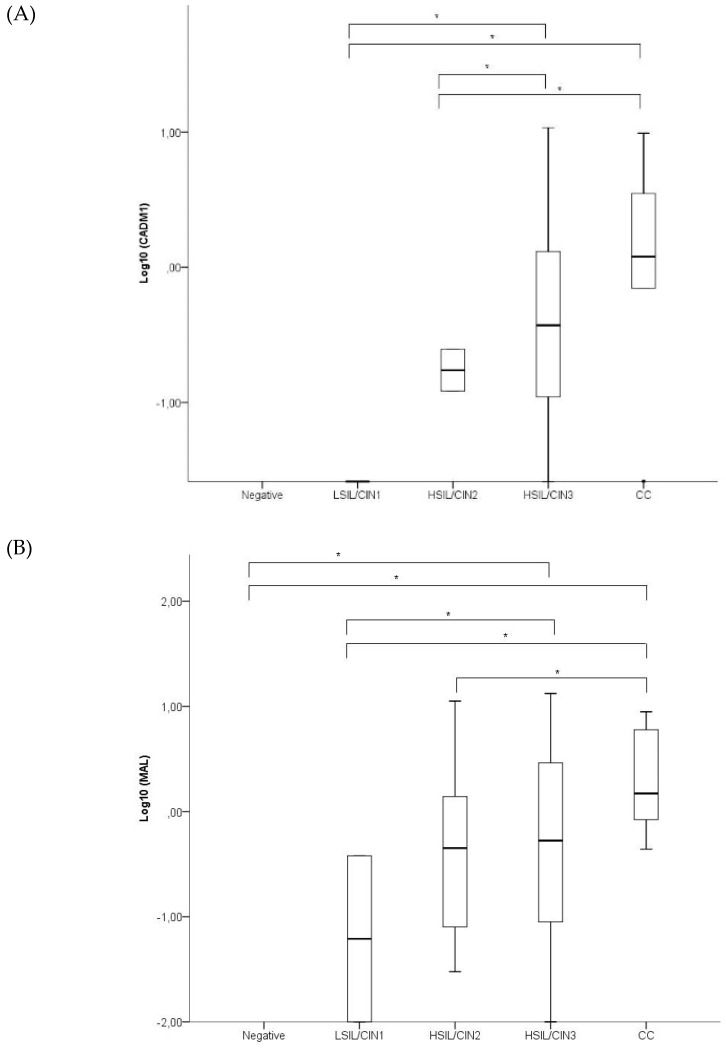

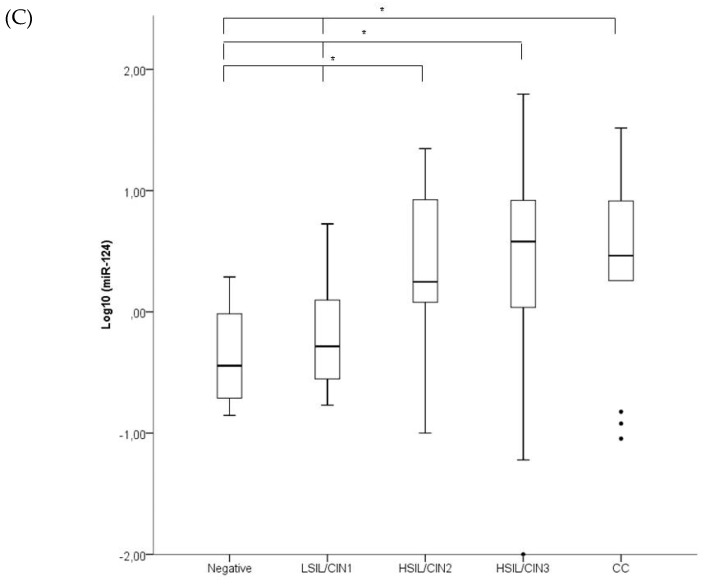
Boxplots of the levels of CADM1 (**A**), MAL (**B**), and miR124 (**C**) expressed as 10log-transformed values, in women with normal, low-grade squamous intraepithelial lesion/cervical intraepithelial neoplasia grade 1 (LSIL/CIN1), high-grade squamous intraepithelial lesion/cervical intraepithelial neoplasia grades 2 (HSIL/CIN2), HSIL/CIN3, and cervical cancer (CC). The black line within the box represents the median; the whiskers represent the minimum and maximum values that lie within 1.5 interquartile ranges from the end of the box. Values outside this range are represented by black dots. * CADM1 showed lower levels of methylation in LSIL/CIN1 samples compared with HSIL/CIN3 and CC (*p* = 0.006 and *p* = 0.031, respectively) and lower levels of methylation in HSIL/CIN2 samples compared with HSIL/CIN3 and CC (*p* = 0.008 and *p* = 0.008, respectively). Methylation levels of the MAL promoter gene were lower in negative and LSIL/CIN1 samples compared with HSIL/CIN3 (*p* = 0.001 and *p* = 0.003, respectively) and CC (*p* = 0.003 and *p* = 0.003, respectively). MAL also showed lower methylation levels in HSIL/CIN2 samples compared with CC (*p* = 0.029). miR124 showed lower levels of expression in negative and LSIL/CIN1 samples compared with HSIL/CIN2 (*p* = 0.001 and *p* = 0.003, respectively), HSIL/CIN3 (*p* < 0.001 and *p*< 0.001, respectively) or CC (*p* = 0.019 and *p* = 0.024, respectively).

Figure 1 shows the ∆∆Cq levels of CADM1, MAL, and miR124 in the different groups. The methylation levels of CADM1, MAL and miR-124 were significantly lower in LSIL samples compared with HSIL/CIN3 and CC. No significant differences were found in methylation levels between negative and LSIL samples for any of the biomarkers. Neither were significant differences found in the methylation levels between HSIL/CIN3 and CC samples. The methylation levels of CADM1 were significantly lower in HSIL/CIN2 compared with HSIL/CIN3 and CC (*p* = 0.008 and 0.008, respectively). HSIL/CIN2 also showed lower methylation levels of MAL compared with CC (*p* = 0.029).

Table 1 shows the methylation status of the MAL, CADM1, and miR-124 promoter genes in the different diagnostic groups as well as the positivity rate for the combination of the three biomarkers. The methylation rates significantly increased with the severity of the lesion for all of the genes. None of the samples from the control group showed methylation of the CADM1 and MAL genes and only one sample showed methylation of miR124. All CC and more than 80% of the HSIL/CIN2-3 lesions showed at least one methylated gene.

Table 2 shows the sensitivity, specificity, PPV, and NPV of the methylation of the different promoter genes for the detection of HSIL/CIN2+ and HSIL/CIN3+ regardless of HPV status. miR124 was the most sensitive marker for the detection of HSIL/CIN2+ and HSIL/CIN3+, whereas CADM1 was the most specific. The sensitivity and specificity of having at least one methylated gene for HSIL/CIN2+ was 84.6% and 74.0%, respectively, and 85.1%, and 43.8% for HSIL/CIN3+. When the three genes were methylated, the specificity for HSIL/CIN2+ was 100%.

### 2.2. Correlation between Methylation Status and the Percentage of the Biopsy Involved by the Lesion

The mean area of the biopsies was 7.9 mm^2^ (SD 5.5 mm^2^). No differences were found between the different diagnostic groups (*p* = 0.395). The mean percentage of the total biopsy area occupied by the squamous epithelium was 50.2mm^2^ (SD 31.9 mm^2^), with no differences observed between the diagnostic groups (51.1% for the normal biopsies, 56.5% for the LSIL/CIN1, 49.9% for the HSIL/CIN2, 46.7% for the HSIL/CIN3 and 83.6% for the CC; *p* = 0.393). The percentage of the squamous epithelium involved by the lesion was 58.9% for the LSIL/CIN1, 77.5% for the HSIL/CIN2, 69.8% for the HSIL/CIN3, and 90.2% for the CC; *p* = 0.423). No correlation has been found between the percentage of squamous epithelium involved by the lesion and the methylation results.

### 2.3. hrHPV Testing and Genotyping and p16 Immunostaining

Within the group of patients with LSIL/CIN1, five out of the 19 (26.3%) samples were positive for hrHPV16/18, nine (9/19; 47.4%) were positive for hrHPV non16-18, four (4/19; 21.1%) were positive for low-risk HPV, and one (5.2%) was negative. All the patients with HSIL/CIN2 were positive for hrHPV, with 50% (15/30) being positive for hrHPV16/18, and 50% (15/30) for hrHPV non16-18. Similarly, all the patients with HSIL/CIN3 were positive for hrHPV, 60% (36/60) being positive for hrHPV16/18 and 40% (24/60) for hrHPV non16-18. Among the group of patients with CC, 78.6% (11/14) were positive for hrHPV16/18, 7.1% (1/14) for hrHPV non16-18, and 14.3% (2/14) were negative for HPV. hrHPV16/18 genotyping showed a sensitivity and specificity for HSIL/CIN2+ of 50.1% and 81.5%, respectively.

p16 immunostaining was positive in 78.9% (15/19) patients with LSIL/CIN1 and in 100% of the HSIL/CIN2, HSIL/CIN3 and CC biopsies.

### 2.4. Combinations of Biomarkers

**Figure 2:**
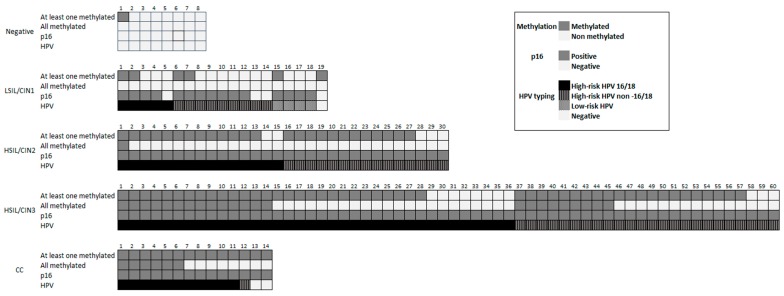
Results of the methylation status of CADM1, MAL, and miR124, p16 immunostaining and HPV testing and genotyping for each case in the different groups included in the study.

Figure 2 shows the results of the methylation status of CADM1, MAL, and miR124, p16 immunostaining and HPV testing and genotyping for each case in the different groups included in the study. 

Table 3 shows the correlation between HPV genotyping and methylation status in the different diagnostic categories. There was no statistically significant correlation between methylation status and HPV genotyping for any of the groups.

The combination of at least one methylated gene and positivity for hrHPV reached a sensitivity of 80.7% and a specificity of 85.1% for HSIL/CIN2+, and the combination of at least one methylated gene and HPV16/18 genotyping showed a sensitivity of 43.3% and a specificity of 92.6%.

## 3. Discussion

In this study, we determined the value of methylation analysis for CADM1, MAL, and miR124 alone, in combination and in association with hrHPV genotyping for the detection of transforming HSIL/CIN in a number of well-characterized cervical biopsies. In keeping with previous reports, in the present study, methylation of CADM1, MAL, and miR124 increased in parallel with the severity of the cervical lesion [16,17]. Interestingly, the combination of at least one methylated gene and positivity for hrHPV achieved a sensitivity of 80.7% and a specificity of 85.1% for the detection of HSIL/CIN2+.

Methylation of CADM1, MAL, and miR124 has been related to the severity and duration of cervical disease [15]. Four studies have recently shown that altered expression of several miRs represents an early event in hrHPV-induced carcinogenesis that is already detectable in SIL/CIN lesions [18,19]. In contrast, the weighted mean methylation frequencies for CADM1 and MAL were highest in transforming CIN lesions [11,20]. Thus, methylation analyses of CADM1, MAL, and miR124 seem to be able to distinguish hrHPV-positive patients with transforming lesions at short-term risk of progression from women with clinically irrelevant infections [11,15,16]. We do not have follow-up data from the patients included in the study, and consequently, we cannot estimate the specific risk of transformation. Nevertheless, our results are in keeping with previous data showing that the methylation status of HSIL/CIN3 is similar to that of CC, meaning that most of them should be considered as truly transforming lesions. In contrast, the methylation status of HSIL/CIN2 shows a dissociated pattern, resembling LSIL/CIN1 for CADM1, but more similar to HSIL/CIN3 and CC for miR124 and MAL, suggesting that HSIL/CIN2 represents probably a heterogeneous group, with some lesions having less risk of progression than HSIL/CIN3. It has also been suggested that HSIL/CIN3 is also a heterogeneous disease, both in terms of the chromosomal aberrations detected and in terms of clinical behavior [10]. In our series, the methylation pattern of HSIL/CIN3 was similar to CC in all the genes analyzed, and although some of the HSIL/CIN3 lesions showed a different methylation pattern, the lack of follow-up data did not allow adequate correlation between methylation patterns and clinical outcomes.

The present study analyzed methylation patterns in histological samples, showing that the result of methylation status is indisputably related to the sample evaluated. The analysis of these biomarkers in HPV-positive women in the present series of biopsies showed a very good diagnostic accuracy for HSIL/CIN2+ (sensitivity and specificity of 84.6% and 74.0%, respectively), which is consistent with current evidence in cytology [11]. We did not test the methylation status in the cytological samples, which would have provided some insight into the possible usefulness of methylation tests in the CC screening. However, a previous study showed that the methylation status of cervical scrapes was highly representative of that of the worst lesion, particularly in the case of HSIL/CIN3 or CC [11].

In the present series, the prevalence of HPV16 and/or 18 increased with the presence of histological abnormalities. These results are similar to previous reports showing that HPV16 and 18 are more frequently associated with transforming infection [21]. In line with previous evidence, HPV16/18 genotyping showed a high specificity (81.5%), which increased to more than 90% when combined with the methylation of at least one biomarker [18]. These results suggest that women with HPV16 and/or 18 infection and methylation of at least one gene should be immediately referred to colposcopy and closely followed in case of negative findings.

The lower anogenital squamous terminology (LAST) standardization project for HPV-associated lesions specifically recommends the use of p16 immunostaining as an adjunct to morphologic assessment of cervical biopsies to increase the sensitivity of HSIL/CIN2+ detection [22]. We used the LAST criteria, and in this study, p16 was negative in all the negative samples and positive in all the HSIL/CIN2-3 lesions. Although controversial results have been published, some studies have suggested that in LSIL/CIN1, p16 immunostaining could have prognostic value [7,23,24]. However, in the present series, no relation was found between p16 immunostaining and the methylation pattern. Thus, our results are in keeping with those of a previous study by our group showing a low value of p16 IHC staining as a marker of progression of LSIL/CIN1 in clinical practice [22].

This study has some limitations. First, changes in the methylation pattern due to the presence of hrHPV in samples without cellular dysplasia were not evaluated. Negative biopsy samples positive for hrHPV were not included in the study, since this category might represent a heterogeneous group including hrHPV infection with no lesions and hrHPV infection with existing, but underdiagnosed, SIL/CIN. Second, the methylation status was evaluated on DNA extracted from the total biopsy and not selectively on the dysplastic cells. However, no correlation was found between the percentage of squamous epithelium involved by the lesion and the methylation results, which indicates a limited potential effect of the different fraction of dysplastic/neoplastic cells in the analysis. Finally, no follow-up data were available, and consequently, the possible relation between methylation and the risk of progression to HSIL/CIN2-3 could not be assessed. Further studies including follow-up data are warranted in order to confirm the relationship between methylation status and the risk of progression.

In conclusion, our study confirms that in a series of well-characterized histological cervical samples, the methylation of CADM1, MAL and miR124 increases with the severity of the lesion, and that the combination of at least one positive biomarker and positivity for hrHPV achieved a sensitivity of 80.7% and a specificity of 85.1% for the diagnosis of HSIL/CIN2-3. Further research including follow-up data is warranted to evaluate the usefulness of these biomarkers for the identification of truly transforming HSIL/CIN.

## 4. Materials and Methods

### 4.1. Study Design and Case Selection

This prospective study was approved by the Institutional Ethical Review Board (HCB/2016/0672, approved on 7 June 2016). All women signed informed consent for inclusion in the study. All patients referred to the Colposcopy Clinic of the Oncological Gynecology Unit of the Hospital Clinic of Barcelona from January 2013–December 2015 were considered eligible for the study. Referral to colposcopy was based on a Pap test result of atypical squamous cell (ASC), with positive hrHPV testing, or atypical glandular cells (AGC), LSIL, HSIL, or CC within 6 months before admission. Of these patients, we selected women who fulfilled the following inclusion criteria: (1) Cervical sample, taken at the referral visit, adequate for Pap-test and hrHPV testing and genotyping; (2) colposcopically directed biopsy and/or endocervical curettage taken concurrently, allowing the study of methylation status and p16 immunohistochemical (IHC) staining in the biopsy specimen; and (3) concordant results between the cervical cytology result and the histological/p16 IHC diagnosis. The following were considered as criteria for exclusion: (1) Previous history of CC, (2) treatment for HSIL/CIN2-3 performed within the previous 3 years; (3) immunosuppression, (4) pregnancy, and (5) low DNA quality isolated for methylation analysis.

The study included five diagnostic groups: (1) Negative for intraepithelial lesion (control group) including women with a negative Pap test, a negative biopsy with negative p16 IHC staining, and a negative hrHPV test result; (2) LSIL/CIN1 including women with a Pap test result of LSIL and a biopsy showing LSIL/CIN1, independently of the p16 IHC staining results; (3) HSIL/CIN2 group including patients with a Pap test result of HSIL and a biopsy confirming HSIL/CIN2, including positive p16 IHC staining; (4) HSIL/CIN3 including patients with a Pap test result of HSIL and a biopsy confirming HSIL/CIN3 with positive p16 IHC staining; and (5) CC group including patients with Pap test result of HSIL or CC and a biopsy confirming CC, independently of the result of the p16 IHC staining.

### 4.2. Patient Management

At the initial visit in the colposcopy clinic, all women underwent cervical sampling using a cytobrush, which was transferred to PreservCyt solution (Hologic, Marlborough, MA, USA). The first part of the sample was used for ThinPrep liquid-based cytology. The residual material was used for hrHPV testing.

Colposcopy was performed in all patients using an Olympus Evis Exera II CV-180 colposcope (Olympus, Barcelona, Spain) after preparing the cervix with 5% acetic acid. Colposcopy findings were described using the criteria of the International Federation for Cervical Pathology and Colposcopy (IFCPC) [25]. In all patients showing an abnormal area in the colposcopic examination, a directed biopsy was performed. In addition, if the transformation zone was not completely visible, endocervical curettage using a Kervokian curette was also performed. A random biopsy was taken from the transformation zone in all the women with a completely visible transformation zone with no colposcopy abnormalities.

### 4.3. Liquid-Based Cytology and Histological Diagnosis

Thin-layer cytology slides were prepared using the Thinprep T2000 slide processor (Hologic, Mississauga, ON, Canada) and stained using the Papanicolaou method. Cytology slides were evaluated by a cytotechnologist and confirmed by an expert pathologist using the revised Bethesda nomenclature [26].

Biopsy specimens were fixed in 10% buffered neutral formalin and paraffin-embedded. Three µm sections were stained with hematoxylin and eosin (H&E), examined by a gynecological pathologist and classified as negative, LSIL/CIN1, HSIL/CIN2, and HSIL/CIN3, or CC. The histological diagnoses were established using morphologic criteria based on the H&E stained sections and the results of the IHC staining for p16, being blinded to knowledge of HPV status or cytology results.

### 4.4. Immunohistochemical Detection of p16

All histological samples were stained with p16 (CINtec Histology kit, cloneE6H4; Roche-Mtm-Laboratories, Heidelberg, Germany) following the manufacturer’s protocol. Briefly, IHC was performed with the Autostainer Link 48 automated system (Dako Co, Carpinteria, CA, USA) using the EnVision system (Dako Co, Carpinteria, CA, USA). Each series included a positive control consisting of a HSIL/CIN3. Cases with a complete absence of p16 staining were classified as negative. The immunostaining was scored as focal when either discontinuous staining of isolated basal cells or any type of staining of superficial and/or suprabasal layers was detected. Diffuse staining was defined as continuous block staining of the basal and suprabasal cells in an area, with both nuclear and cytoplasmic reaction, independently of whether the superficial cells of the squamous epithelium were stained or not [27]. Only diffuse staining was considered a positive reaction. The histological diagnosis of negative for intraepithelial lesion required a negative result for p16 IHC staining. A p16 positive staining was required for the diagnosis of HSIL/CIN2 and HSIL/CIN3. The diagnoses of LSIL and CC were independent of the results of the p16 IHC.

### 4.5. Nucleic-Acid Isolation from Histological Samples

DNA extraction was performed on formalin-fixed paraffin-embedded histological tissue from colposcopically-directed biopsies or endocervical curettages. The samples were serially sectioned on a microtome. The first and last sections were stained with H&E for histological confirmation of the diagnosis. In-between sections were collected in RNAase-free reaction tubes for DNA isolation (sandwich cutting technique).

Sectioning and sample preparation were carried out with the highest safety measures to avoid contamination and cross-contamination. Paraffin blocks lacking tissue were cut in between the patient samples as controls to ensure the absence of contamination. None of these control samples were positive in the HPV PCR assay, indicating adequate avoidance of contamination.

DNA was extracted by overnight incubation in 20 µL of proteinase K solution (1 mg/mL) at 70 °C. Subsequently, proteinase K was heat-inactivated by incubation of the sections at 95 °C for 10 min, and samples were spun down and cooled down at −20 °C for 1–2 min. DNA was isolated using a commercially available kit (QIAamp DNA minikit; Qiagen, Hilden, Germany), according to the manufacturer’s instructions, and 10 μL of isolated DNA was used for PCR amplification. DNA yields were quantified spectrophotometrically using the NanoDrop ND–1000 (Thermo Scientific NanoDrop, Wilmington, DE, USA).

### 4.6. hrHPV Genotyping of Histological Samples

For hrHPV detection, DNA isolated from cervical biopsies was used directly for amplification by broad-spectrum primers that amplify a 65 bp region of the L1 gene. The amplification products were detected by the HPV SPF10 PCR DNA enzyme immunoassay (DEIA) system (Labo Bio-medical Products, Rijswijk, the Netherlands), which detects at least 54 different HPV genotypes. DEIA-positive SPF10 amplimers were used to identify the HPV genotype by reverse hybridization on a line probe assay (SPF10 HPV LIPA version 1, Labo Bio-medical Products B.V., Rijswijk, The Netherlands), which detects 25 HPV genotypes (6, 11, 16, 18, 31, 33, 34, 35, 39, 40, 42, 43, 44, 45, 51, 52, 53, 56, 58, 59, 66, 68, 70, and 74). Each run contained negative and internal and external positive controls to monitor for the efficiency of DNA isolation, PCR amplification, hybridization, and genotyping procedures. Based on epidemiological analyses, 15 of these HPV types were regarded as high risk (16, 18, 31, 33, 35, 39, 45, 51, 52, 56, 58, 59, 68, 53, 66), and the others were classified as low risk (6, 11, 34, 40, 42, 43, 44, 54, 70, 74).

### 4.7. Bisulfite Treatment and Quantitative Methylation Specific PCR of Histological Samples

Bisulfite treatment was performed using 250 ng of genomic DNA with the EZ DNA Methylation KitTM (Zymo Research, Orange, CA, USA) as described elsewhere [16]. The bisulfite conversion protocol described for the cervical scrapes was adapted to the biopsies with an additional pretreatment incubation for 10 min at 95 °C, and denaturation was performed at 95 °C for 20 min.

After bisulfite treatment, methylation of the promoter regions of the cellular genes CADM1-m18, MAL-m1, and hsa-miR-124-2 was tested using the prototype PreCursor-M kit (Self-Screen BV, Amsterdam, The Netherlands). This is a quantitative multiplex methylation-specific PCR (qMSP), based on the TaqMan technology. Samples were run in single, separate reactions on an ABI 7500 Fast Real-time PCR system (Applied Biosystems, Foster City, CA, USA) following the manufacturer’s instructions. In addition, a methylation-independent β-actin was included in the kit as a sample reference to determine the total amount of converted human DNA present in the reaction. To assure sample quality, samples with Cq-values for β-actin >32 were considered of poor DNA quality (invalid) and excluded from the analysis.

### 4.8. Evaluation of the Percentage of the Biopsy Involved by the Lesion

In order to evaluate the potential different fraction of dysplastic/neoplastic cells in the samples analyzed, the percentage of the biopsy involved by the lesion was evaluated in all cases. The morphometric evaluation was performed by scanning all H&E stained slides with a whole slide scanner Ventana iScan HT (Roche Diagnostics, Sant Cugat, Spain) at a magnification of 20×. The high-resolution digital images of the tissue sections were measured using the Virtuoso viewer (Roche, Sant Cugat, Spain), which includes measuring tools. The total area of the biopsy, the total area occupied by squamous epithelium, and the area involved by the dysplastic epithelium were measured in each biopsy.

### 4.9. Statistical Methods

Categorical variables are presented as absolute number and percentages and compared using the χ2 or Fisher exact test. Continuous variables are presented as mean and standard deviation (SD) and were compared using the analysis of variance test. The quantification cycle (Cq-value) was determined for each target (CADM1, MAL, and miR124) using the ∆∆Cq method [16]. This method shows the relative difference between each marker and β-actin in a sample compared with the internal calibrator (Calibrator PreCursor-M kit Self-screen B.V, molecular assays, Amsterdam, The Netherlands) according to the manufacturer’s recommendations.

For calculations of methylation status, a sample was scored as methylation-positive for a specific target when the target gene/β-actin ratio was above a fixed calculated threshold value of the respective target. For each gene, the threshold was the upper limit of the 99% confidence interval (CI) of the mean ratio of all histologically normal samples. Overall, a sample was considered to be methylation-positive when at least one target was above its threshold. The frequencies of CADM1, MAL, and miR124 methylation positive cervical biopsies in relation to their histological diagnosis were calculated. The 95% CIs were determined for each proportion of methylated samples. The proportions of overall methylation-positive samples, HPV genotyping, and p16 immunostaining per histological diagnosis were compared using the Mann-Whitney U test. *p* values less than 0.05 were considered significant. The efficacy of gene methylation for the diagnosis of HSIL/CIN2+ was evaluated as sensitivity and specificity.

The sensitivity and specificity and positive and negative predictive values (PPV and NPV, respectively) were calculated for the combination of CADM1, MAL, and miR124 promoter methylation status and HPV genotyping. The endpoints were the histological diagnosis of HSIL/CIN2+ and HSIL/CIN3+. Calculations were performed using SPSS version 22.0 software (SPSS, Inc., Chicago, IL, USA).

## Figures and Tables

**Table 1:** Positivity rates for MAL, CADM1, and miR-124 methylation and combinations of at least one, two, or three methylated biomarkers in the different diagnostic groups. LSIL/CIN1: Low-grade squamous intraepithelial lesion/cervical intraepithelial neoplasia grade 1; HSIL/CIN2: High-grade squamous intraepithelial lesion/cervical intraepithelial neoplasia grade 2; HSIL/CIN3: High-grade squamous intraepithelial lesion/cervical intraepithelial neoplasia grade 3; CC: Cervical cancer.

Biomarker	Negative (*n* = 8)		LSIL/CIN1 (*n* = 19)		HSIL/CIN2 (*n* = 30)		HSIL/CIN3 (*n* = 60)		CC (*n* = 14)		*p*
	*n*	(%)	*n*	(%)	*n*	(%)	*n*	(%)	*n*	(%)	
CADM1	0	(0.0)	2	(10.5)	2	(6.6)	31	(51.6)	8	(57.4)	<0.001
MAL	0	(0.0)	2	(10.5)	17	(56.6)	36	(60.0)	13	(92.8)	<0.001
miR124	1	(12.5)	3	(15.7)	20	(66.6)	41	(68.3)	11	(78.5)	<0.001
At least one methylated gene	1	(12.5)	6	(31.5)	25	(83.3)	49	(81.6)	14	(100)	<0.001
At least two methylated genes	0	(0.0)	1	(5.2)	13	(43.3)	36	(60.0)	12	(85.7)	<0.001
Three methylated genes	0	(0.0)	0	(0.0)	1	(3.3)	23	(38.3)	6	(42.8)	<0.001

**Table 2:** Sensitivity, specificity, positive, and negative predictive values (PPV and NPV, respectively) of the methylation of MAL, CADM1, or miR-124, and of at least one, two, or the three genes for HSIL/CIN2+ and HSIL/CIN3+. HSIL/CIN2+ includes high-grade squamous intraepithelial lesion/cervical intraepithelial neoplasia grade 2 (HSIL/CIN2), HSIL/CIN3 and cervical cancer (CC). HSIL/CIN3+ includes HSIL/CIN3 and CC.

	Sensitivity	Specificity	PPV	PNV
**HSIL/CIN2+**				
CADM1	39.4	92.5	95.3	28.4
MAL	63.4	92.5	97.0	39.0
miR124	69.2	85.1	94.7	41.8
At least one methylated gene	84.6	74.0	92.6	55.0
Two methylated genes	58.6	96.2	98.3	37.6
Three methylated genes	28.8	100.0	100.0	26.7
**HSIL/CIN3+**				
CADM1	52.7	92.9	90.6	60.2
MAL	66.2	56.7	72.0	60.3
miR124	70.2	57.8	68.4	60.0
At least one methylated gene	85.1	43.8	66.3	69.4
Two methylated genes	64.8	75.4	77.4	62.3
Three methylated genes	39.1	98.2	96.6	55.4

**Table 3:** Correlation between HPV genotyping and methylation of at least one biomarker in the different diagnostic groups. HPV: Human papillomavirus; lrHPV: Low-risk HPV; LSIL/CIN1: Low-grade squamous intraepithelial lesion/cervical intraepithelial neoplasia grade 1; HSIL/CIN2: High-grade squamous intraepithelial lesion/cervical intraepithelial neoplasia grade 2; HSIL/CIN3: High-grade squamous intraepithelial lesion/cervical intraepithelial neoplasia grade 3; CC: Cervical cancer.

Histological Diagnosis	Methylation Status	HPV Genotyping						
		Negative/lrHPV		HPV No 16/18		HPV16/18		*p*
		*n*	(%)	*n*	(%)	*n*	(%)	
**Negative**								-
	Positive	1	(100.0)	-	(-)	-	(-)	
	Negative	7	(100.0)	-	(-)	-	(-)	
**LSIL/CIN1**								0.707
	Positive	2	(33.3)	2	(33.3)	2	(33.3)	
	Negative	3	(23.1)	7	(53.8)	3	(23.1)	
**HSIL/CIN2**								1
	Positive	0	(0.0)	12	(48.0)	13	(52.0)	
	Negative	0	(0.0)	3	(60.0)	2	(40.0)	
**HSIL/CIN3**								0.500
	Positive	0	(0.0)	21	(42.9)	28	(57.1)	
	Negative	0	(0.0)	3	(27.3)	8	(72.7)	
**CC**								-
	Positive	2	(14.3)	1	(7.1)	11	(78.6)	
	Negative	0	(0.0)	0	(0.0)	0	(0.0)	
